# Chromosome-scale genome assembly of oil-tea tree *Camellia crapnelliana*

**DOI:** 10.1038/s41597-024-03459-x

**Published:** 2024-06-07

**Authors:** Fen Zhang, Li-ying Feng, Pei-fan Lin, Ju-jin Jia, Li-zhi Gao

**Affiliations:** Engineering Research Center for Selecting and Breeding New Tropical Crop Varieties, Ministry of Education; Tropical Biodiversity and Genomics Research Center, Hainan University, Haikou, 570228 China

**Keywords:** Plant evolution, Conservation biology, Genetic variation, Phylogenetics, Plant ecology

## Abstract

*Camellia crapnelliana* Tutch., belonging to the Theaceae family, is an excellent landscape tree species with high ornamental values. It is particularly an important woody oil-bearing plant species with high ecological, economic, and medicinal values. Here, we first report the chromosome-scale reference genome of *C. crapnelliana* with integrated technologies of SMRT, Hi-C and Illumina sequencing platforms. The genome assembly had a total length of ~2.94 Gb with contig N50 of ~67.5 Mb, and ~96.34% of contigs were assigned to 15 chromosomes. In total, we predicted 37,390 protein-coding genes, ~99.00% of which could be functionally annotated. The chromosome-scale genome of *C. crapnelliana* will become valuable resources for understanding the genetic basis of the fatty acid biosynthesis, and greatly facilitate the exploration and conservation of *C. crapnelliana*.

## Background & Summary

As one of the four largest woody oil plants in the world, oil-tea camellia trees are a collective term for a group of *Camellia* species of highly economic values^1^. In China, oil-tea camellia trees have a long history of cultivation, which are mainly distributed in the south of the lower reaches of the Yangtze River^2,3^. There are approximately 50 species of such oil-tea camellia trees belonging to the family Theaceae^4^. *C. oleifera*, *C. chekiangoleosa*, *C. crapnelliana* and *C. vietnamensis*^1,3^ are commonly cultivated. They are woody, oil-bearing tree species with a high content of seed oil that is widely processed into skin and health care products and especially edible oil^4^. Camellia oil is remarkably rich in polyphenols, saponins, and other healthy components and free of cholesterol, erucic acid, and other harmful components^5^. Thus, the oil has extremely high nutritional and health-beneficial values and has strong market competitiveness and wide market prospects^6^. The content of unsaturated fatty acids in the edible oil is quite high, reaching approximately 90%, and the content of oleic acid can be approximately 87%^5^. Tea oil is therefore referred to as “Oriental olive oil”^7^, which has both health-beneficial and medicinal values^8^.

**Fig. 1:**
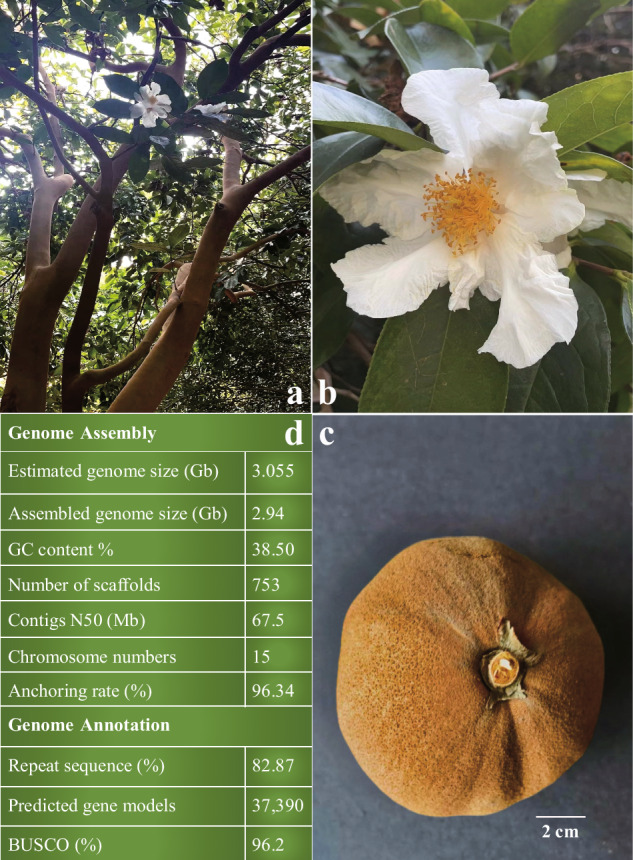
Summary of genome assembly and plant features of *C. crapnelliana*. Plant tree (**a**), flower (**b**), fruit (**c**) and genome assembly statistics (**d**) of *C. crapnelliana*.

Among these oil-tea camellia species, *C. crapnelliana*, which belongs to Sect. *Furfuracea* and is naturally distributed in Hong Kong, southern Guangxi, northern Fujian, southern Zhejiang and Jiangxi provinces, China, was listed as China’s second-class protected plant species and recorded in the China Plant Red Data Book (CPRDB) as early as 1992^9^. As an excellent garden greening species with the largest flowers and fruits (Fig. 1) in the genus *Camellia*, it has great potential for the industrial development as an oilseed plant^10^. Most recently, several chromosome-level tea tree genomes became publicly available^11–16^, but oil-tea camellia tree genome information is still quite limited^11–16^. Many efforts have been put on the regulation of the fatty acid biosynthesis in many plants^17–29^, however, in-depth understanding about the molecular basis and evolution of the fatty acid biosynthesis in *C. crapnelliana* largely rely on a high-quality reference genome.

**Fig. 2:**
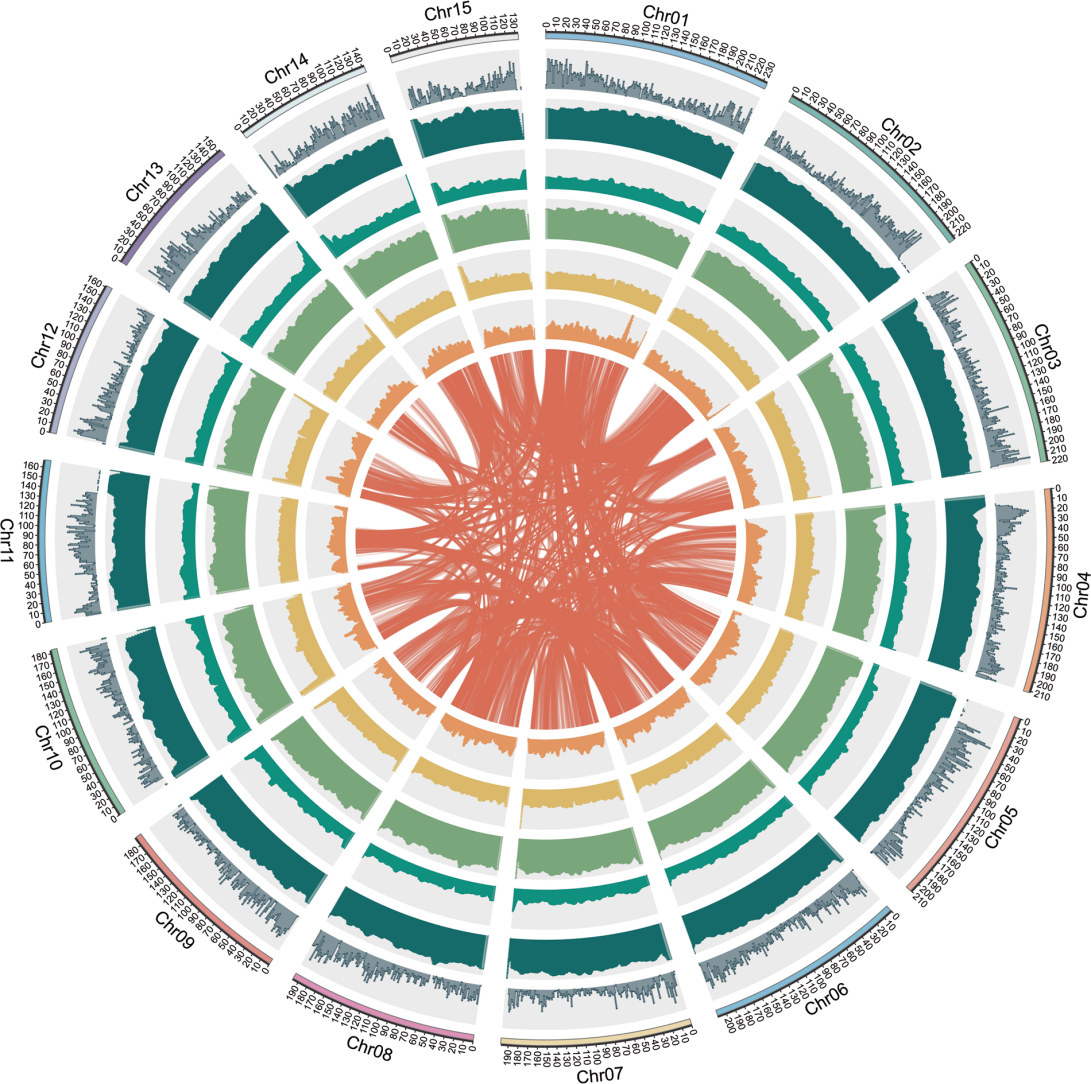
Overview of features of the *C. crapnelliana* genome. The outermost layer represents 15 pseudo-chromosomes of the *C. crapnelliana* genome (scale mark = 1 Mb), and the second to seventh circles symbolize the density of protein-coding genes, repeat sequence density, GC content, total TEs, *Gypsy*-like element distribution, and *Copia*-like element distribution. The innermost track indicates genomic synteny among the chromosomes.

In this study, we constructed and annotated a high-quality chromosome-level reference genome of *C. crapnelliana* using integrated sequencing data (~71 × PacBio HiFi reads and ~140 × Hi-C reads) (Fig. 2). *K-mer* analysis showed that the genome size of *C. crapnelliana* was estimated to be ~3.055 Gb, with a repeat sequence proportion of 76.76% (Supplementary Table S1). The final assembled genome was ~2.94 Gb, with contig N50 of ~67.50 Mb (Fig. 1d). Based on the karyotype of the species (2n = 30)^30^, approximately ~96.34% of the contig reads were anchored to 15 pseudochromosomes. A total of 37,390 protein-coding genes were predicted, of which 99.00% were functionally annotated. In addition, 176 miRNAs, 7,988 rRNAs, 857 tRNAs, and 485 snRNAs in the *C. crapnelliana* genome were annotated. The high-quality chromosome-level genome assembly of this oil-tea *Camellia* species will greatly help to enhance the functional analysis of novel genes towards oil quality and yield improvement, and augment its wild resources conservation and utilization in the future.

## Methods

### Plant materials, sample collection, and sequencing

For genomic DNA extraction, young healthy leaves of *C. crapnelliana* were collected from South China National Botanical Garden, Guangzhou, China. Sampled leaves were immediately flash-frozen in liquid nitrogen and stored at −80 °C until further use. High molecular weight genomic DNAs (gDNAs) were extracted from leaves using improved CTAB method^31^ and evaluated using NanoDrop One spectrophotometer (NanoDrop Technologies, Wilmington, DE) and Qubit 3.0 Fluorometer (Life Technologies, Carlsbad, CA, USA). For the genome survey, the paired-end (PE 150 bp) library was generated using the Illumina TruSeq DNA Nano Preparation Kit (Illumina, San Diego, CA, USA), and the library was sequenced on an Illumina HiSeq. 2500 platform following the manufacturer’s instructions. As a result of Illumina sequencing, we obtained ~173.51 Gb of Illumina paired-end reads (Supplementary Table S2). The Pacbio HiFi sequencing was then performed on the PacBio Sequel II platform (Pacific Biosciences, CA, USA), according to the manufacturer’s instructions. We obtained ~212.87 Gb HiFi reads with an average read length of ~19,232.96 bp, which covered about 71 × of the *C*. *crapnelliana* genome (Supplementary Table S2). For Hi-C sequencing, formaldehyde was used for crosslinking the fresh leaves, and the crosslinking reaction was terminated using glycine solution. Subsequently, the Hi-C library was constructed based on the instructions and sequenced on the Illumina platform (Annoroad Gene Technology Co., Ltd), and ~429.88 Gb raw reads were generated (Supplementary Table S2). The young leaves, flowers, young shoots, and seed kernels were collected for transcriptome sequencing. These tissue samples were rinsed using ddH_2_O and stored at −80 °C until use after snap-freeze using liquid nitrogen with three biological replicates. Total RNA extraction was performed using the RNeasy Plant Mini Kit (Qiagen, Hilden, Germany). A cDNA library was built following the instructions, followed by paired-end sequencing on the NovaSeq platform (Illumina). A total of ~30.00 Gb RNA-seq reads were obtained to assist the subsequent analysis of the *C. crapnelliana* genome.

### Chromosome-level genome assembly

Genome size of *C*. *crapnelliana* was estimated from Hi-C data using *k-mer* frequency analysis. Jellyfish v2.3.0^32^ was first applied to extracting and counting canonical *k*-mer at k = 21. Subsequently, findGSE v1.94^33^ was used to estimate the genome size from *k-mer* count data with parameters of “-k = 21”. As a result, we estimated the genome size of *C. crapnelliana* to be ~3.055 Gb (Supplementary Table S1). The PacBio HiFi reads were *de novo* assembled by using hifiasm v0.16.1^34^ with default parameters. The genome assembly had a total size of ~2.94 Gb, containing 816 contigs with N50 sizes of ~67.5 Mb (Supplementary Table S3). The cleaned Hi-C reads were mapped to the corresponding contigs using Juicer v1.9.9^35^. The unique mapped reads were taken as input for 3D-DNA pipeline v180114^36^ with parameters “-r 0” and then sorted and corrected manually using JuicerBox v1.11.08^37^. The fifteen pseudochromosomes were identified by distinct interaction signals in the Hi-C interaction heatmap (Supplementary Fig. S1), and the final assembled genome length was ~2.94 Gb (Figs. 1d, 2), with a scaffold N50 of ~67.50 Mb, containing ~96.34% of the assembled contigs for *C. crapnelliana* (Supplementary Table S4), accounting for ~96.34% of the estimated genome size based on the *k-mer* analysis (Supplementary Table S1). Compared to the ten other genome assemblies publicly available in the genus *Camellia*, the chromosome-level genome assembly of *C*. *crapnelliana* obtained in this study showed remarkable sequence continuity and genome completeness (Supplementary Table S5).

### Genome annotation and functional prediction

The repetitive elements in the *C. crapnelliana* genome were identified by combining *de novo* and homology-based approaches. Tandem repeat sequences were annotated using Tandem Repeat Finder (TRF v4.09)^38^ with default parameters. A total of six types (mono- to hexa-nucleotides) of simple sequence repeats (SSRs) were identified using the MISA (MIcroSAtellite)^39^ identification tool with default parameters. For *de novo*-based searches, RepeatModeler v2.0.2a^40^, LTR_FINDER v1.07^41^, LTRharvest v1.5.9^42^, and LTR_retriever v2.9.1^43^ were applied for constructing *de novo* repeat libraries, by which RepeatMasker v4.1.3-p1^44^ was employed to detect repeat sequences. For homology-based searches, we employed RepeatMasker v4.1.3-p1^44^ against a known repeat library, Repbase v.19.06^45^. As a result, a total of ~2.44 Gb of repetitive elements occupying ~82.87% of the *C. crapnelliana* genome were annotated (Fig. 2; Supplementary Table S6). Most of these repeats were long terminal repeat (LTR) retrotransposons (~63.24%) of the genome; Supplementary Table S6). The DNA, LINE, and SINE classes accounted for ~10.84%, ~4.19%, and ~0.13% of the genome, respectively (Fig. 2; Supplementary Table S6). Additionally, tRNAscan-SE v2.0^46^ software was used to predict tRNA genes. The rRNA, miRNA, and snRNA were predicted using INFERNAL (v1.1.2)^47^ software through searches against the Rfam database v9.1^48^. Finally, we annotated 176 miRNAs, 7,988 rRNAs, 857 tRNAs, and 485 snRNAs in the *C*. *crapnelliana* genome (Supplementary Table S7).

To annotate protein-coding genes in the *C*. *crapnelliana* genome, gene models were obtained by combining the three approaches of *ab initio* gene predictions, homology-based predictions, and transcriptome-based predictions. The *ab initio* prediction was performed by AUGUSTUS v3.3.2^49^, SNAP^50^ v2013-11-29, GeneMark-ES/ET^51^, GlimmerHMM^52^ v3.02. For homology-based prediction, the Exonerate^53^ v2.2.0 program was used to search against the protein sequences of *Actinidia chinensis*^54^, *Arabidopsis thaliana*^55^, *Beta vulgaris*^56^, *C. oleifera*^26^, DASZ^14^, *C. sinensis* var. *assamica* YK10^11^, *Olea europaea*^57^, *C. chekiangoleosa*^58^, *C. lanceoleosa*^59^, *Vitis vinifera*^60,61^, and *Oryza sativa*^55^ genomes. For transcriptome-based prediction, Trinity v2.15.1^62^ was used for assembling transcripts based on RNA-seq data, and PASA^63^ v2.5.2 software was employed for gene structure prediction based on transcriptome assemblies. Additionally, HISAT2 v2.2.1^64^ was employed for RNA-seq reads mapping onto the genome, and StringTie^65^ v2.2.1 was used for the generation of transcript structure. The assembled transcripts were subsequently used for ORF (open reading frame) prediction using TransDecoder v5.5.0. All predicted gene structures were integrated into a consensus set with EVidenceModeler (EVM v2.0.0)^66^. Finally, 37,390 gene models were predicted after integrating the results of the three aforementioned methods.

For the functional annotation of protein-coding genes, we aligned the predicted protein-coding gene sequences against public functional databases using BLAST v2.11.0^67^ (e-value < 1e-5), including Swiss-Prot^68^, NR^69^, KEGG, and KOG^70^. Gene Ontology (GO) was performed using InterProScan v5.55-88.0^71,72^ (Supplementary Fig. S2). As a result, a total of 37,015 protein-coding genes were annotated for *C*. *crapnelliana*, accounting for ~99.00% of all predicted genes (Supplementary Table S10). Predicted gene models were comparable to the fifteen other species in aspects such as gene number, average gene length, average CDS length, average exons per gene, average introns per gene, average exon length, and average intron length (Supplementary Table S11).

### Genome synteny analysis and the detection of whole-genome duplication (WGD)

**Fig. 3:**
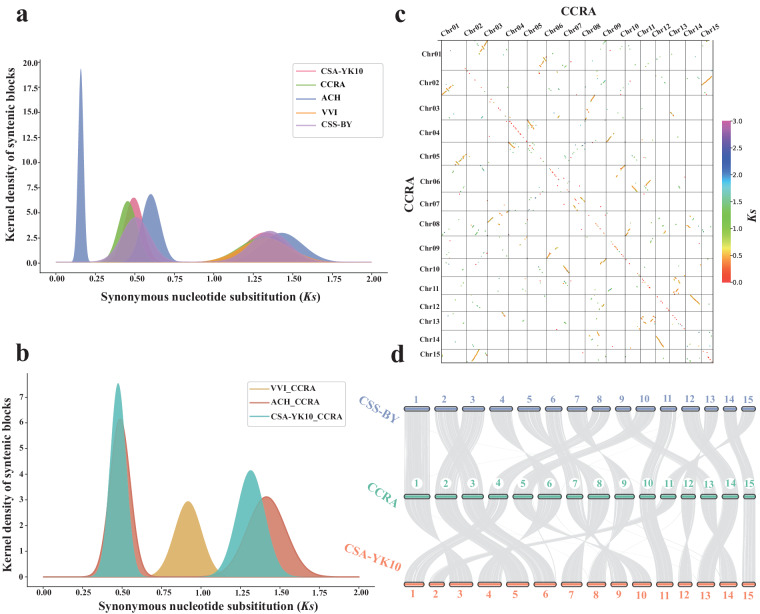
Detection of whole genome duplication (WGD) and genomic synteny analysis between the *C. crapnelliana* and *C. sinensis* genomes. (**a**,**b**) Distribution of *Ks* values in *Actinidia chinensis* (ACH)*, C. sinensis* var*. assamica* (CSA-YK10)*, C. sinensis* var*. sinensis* (CSS-BY), *Vitis vinifera* (VVI), and *C. crapnelliana* (CCRA), which represents the Gaussian fit of the raw *Ks* counts from paralogs. (**c**) Synteny blocks of the *C. crapnelliana* genome. The axes refer to different chromosomes, and genomic synteny blocks represent the WGD event. (**d**) Diagram showing genomic collinearity among the *C. sinensis* var*. sinensis* (CSS-BY), *C. sinensis* var*. assamica* (CSA-YK10), and *C. crapnelliana* (CCRA) genomes.

The WGD analyses were performed using all paralogous gene pairs. MAFFT v7.520^73^ was employed to conduct sequence alignment. The protein sequence alignment was converted into a codon alignment using PAL2NAL v14. Finally, the *Ka* and *Ks* values were obtained using yn00 v4.10.0 of PAML^74^ with the Nei-Gojobori (NG) method. Genes with *Ks* < 0.1 were excluded from further analyses (Supplementary Table S12)^75^. WGDI was adopted to mark the *Ks* on the syntenic block with different colors. The PeaksFit (−pf), Kspeaks (−kp), and KsFigures (−kf) tools of WGDI were used to illustrate the *Ks* density. The *C. crapnelliana* genome exhibited two peaks in the *Ks* density plot (Fig. 3a,b). Our results showed that the occurrence of two polyploidization events in the *C. crapnelliana* genome, including the ancient WGT (γ) event that occurred in grape and eudicots^60,61^, the other WGD (β) event shared with *A. chinensis* and other Theaceae species^11,54,76^ (Fig. 3a,b). We finally verified the occurrence of two WGD events in the *C. crapnelliana* genome by combining genomic synteny analysis and dot plots (Fig. 3c,d) of *C. crapnelliana*.

## Data Records

The MGI short reads, PacBio HiFi long-reads, Hi-C reads, genome assembly and annotation data were deposited in the NCBI SRA database under accession number SRR28825902-SRR28825908^77–83^ and National Genomics Data Center (NGDC)^84^, Beijing Institute of Genomics, the Chinese Academy of Sciences/China National Center for Bioinformation with BioProject accession numbers PRJCA022516^85^. The genome sequencing data were deposited in the Genome Sequence Archive (GSA) of NGDC under Accession Numbers CRA014272^86^. The genome assembly has been deposited in DDBJ/ENA/GenBank under the accession number JBDORG000000000^87^. The genome assembly and annotation data were deposited in Genome Assembly Sequences and Annotations (GWH) of NGDC under accession number GWHERAW00000000^88^. The genome assembly and annotation were also deposited at the figshare database^89^.

## Technical Validation

### Assessment of the genome assembly

The completeness of the assembled genome was evaluated using BWA (v0.7.17)^90^ and Benchmarking Universal Single-Copy Orthologs (BUSCO, v5.4.4)^91^ with the embryophyta_odb10 lineage dataset. Approximately, ~99.67% of the Illumina short reads were aligned to the genome, of which ~93.97% of reads were properly mapped. The BUSCO analysis showed that the assembled genome sequences contained 1,600 (~99.2%) complete BUSCOs, including 1,405 (~87.1%) single-copy BUSCOs, 195 (~12.1%) duplicated BUSCOs, and 8 (~0.5%) fragmented BUSCOs (Supplementary Table S8).

### Assessment of the gene annotation

The annotated and integrated proteins were also evaluated using BUSCO v5.4.4^91^ with the lineage dataset embryophyte_odb10. Briefly, the proportion of complete core gene coverage was ~96.2% (including ~87.3% single-copy genes and ~8.9% duplicated genes), and there were only a few fragmented (~1.4%) and missing (~2.4%) genes (Supplementary Table S9), indicating high-quality annotation of the predicted gene models.

### Genome synteny analysis and WGD detection

The Whole-Genome Duplication Integrated analysis tool (WGDI v0.6.5)^92^ was used for the detection of WGDs, intragenomic collinearity analysis, *Ks* estimation and peak fitting in *C. crapnelliana* (CCRA), *C. sinensis* var*. assamica*^11^(CSA-YK10)*, C. sinensis* var. *sinensis*^12^(CSS-BY), *Actinidia chinensis*^54^(ACH), and *Vitis vinifera*^60,61^(VVI). JCVI^93^ v1.3.6 was further employed to draw the collinearity diagram across these species.

### Supplementary information

Supplementary Figure

Supplementary Table

## Data Availability

All software and pipelines were executed according to the manual and protocols of the published bioinformatic tools. All software used in this work is publicly available, with versions and parameters clearly described in Methods. If no detailed parameters were mentioned for a software, the default parameters suggested by the developer were used. No custom code was used during this study for the curation and/or validation of the datasets.
