# Supplementary material for: Chromosome-scale genome assembly of oil-tea tree Camellia crapnelliana
Source: Sci Data. 2024 Jun 7;11:599. doi: 10.1038/s41597-024-03459-x (PMC11161624; doi:10.1038/s41597-024-03459-x)
Supplement: Supplementary file 1 — Supplementary Figure [file 41597_2024_3459_MOESM1_ESM.docx]

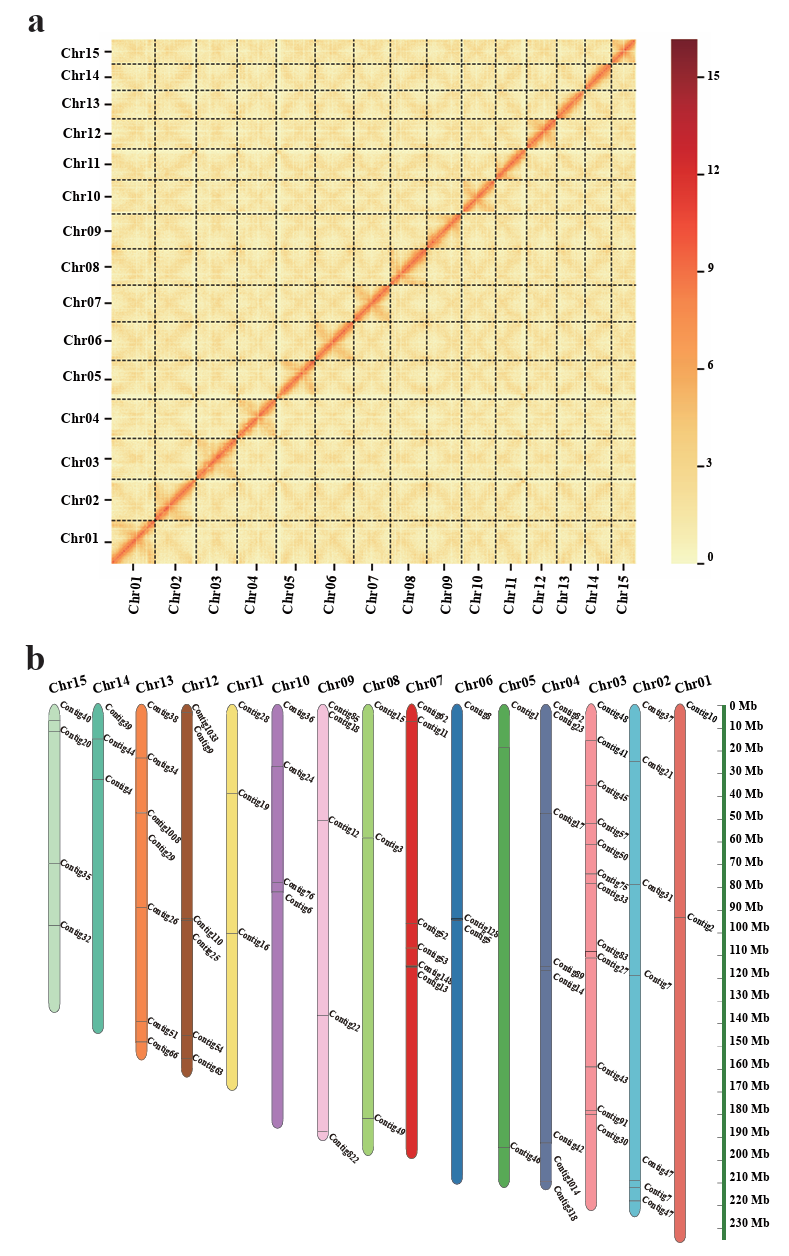


**Supplementary Fig. S1 Genome-wide Hi-C interaction map and chromosome karyogram of** ***C. crapnelliana*.** (**a**) The heat map shows the intensity signals of Hi-C chromosome interaction；(**b**) The chromosome karyogram of the *C. crapnelliana* genome.


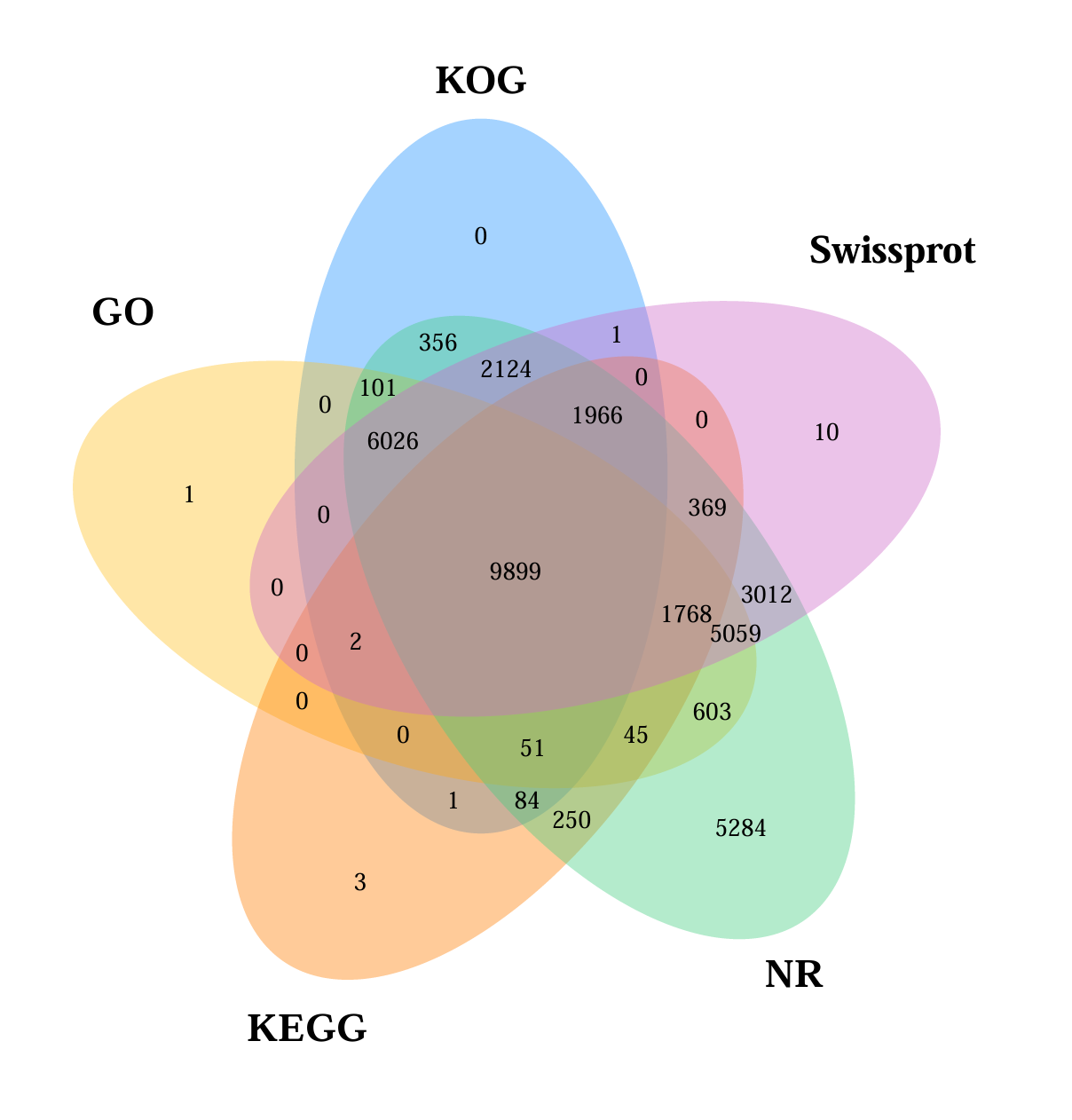


**Supplementary Fig. S2 Venn diagram of the number of genes from *C*. *crapnelliana* with homology or functional classification.**
